# The Medical Student and His Work

**Published:** 1910-04-23

**Authors:** Dyce Duckworth


### SPECIAL ARTICLE.

### THE MEDICAL STUDENT AND HIS WORK.

An Interview with SIR DYCE DUCKWORTH, Bart., M.D., LL.D., F.R.C.P.

Sir Dyce Duckworth is a Liverpool man. His connection with the Liverpool school dates back to the late 'fifties of the last century, when he studied chemistry and materia medica there, and attended the practice at the Royal Infirmary. His recollections of those early days are doubly interesting, because to-day he is still—and, we trust, will for many years yet remain—an authority; a worker who has not, like Entellus, laid aside his caestus and his art content with victories so far achieved, but is yet actively running in the race with a younger generation and keeping his position without undue exertion. Perhaps his success has been largely due to the thoroughness with which he started and prosecuted his studies, and, as he himself admits, in the preliminary sciences at least he had some advantages which the modern medical student misses. "Botany and materia medica were more practically taught then than they are to-day," he says, with a spark of assertiveness in his voice. "I am sure of it," and then he goes on to chat of his old-time infirmary experiences, both in Liverpool and Edinburgh. "I recollect how one of my first attempts at practical work was assisting to dress a case after amputation of the thigh, and how, some days later, I saw the stump streaming with fœtid pus. That was the usual thing in those days. I was pupil under Mr. Edward Bickersteth, who was one of the finest operating surgeons I have ever seen. Professor Syme told me he was the best surgeon in England in his day. The Edinburgh school in those days was one of the greatest schools of medicine. It had a brilliant collection of teachers, Christison, Goodsir, Playfair, Syme, Simpson, and others junior such as Matthews Duncan, Turner, and Cleland. I went to Edinburgh and worked there. I was dresser to Syme when Lister was assistant surgeon, and a clinical clerk with Hughes Bennett and Laycock. I was on duty under Professor Syme on the occasion of his performing the old operation on the patient with gluteal aneurism, one which has been much commented and reported upon.* Later I became house physician to Bennett and Laycock. Great changes were going on, and it was unavoidable that one should instinctively compare Edinburgh with Liverpool. In both the nursing was bad. Good nurses as we now know them were unknown, but those we had—good old women of the charwoman class—were kind and attentive to the patients. We had few male nurses. There was a great deal of fever—as much typhus as typhoid—and, of course, practically no isolation: cases of all kinds were mixed in the medical wards. Thus I recollect in 1859 coming across a case of small-pox in a general ward in a Dublin hospital in the next bed to a patient suffering from Bright's disease. Typhus fever did not spread much, but sometimes the sisters and nurses took it. The early study of thermometry with unregistering thermometers in this fever was especially risky. I once had charge of a typhus ward in Edinburgh with 17 cases at a time."

### A Note on Hobbies.

Sir Dyce believes in study-travel, and has taken full advantage of his journeys abroad to get into touch with foreign hospitals and foreign methods. He regards French and Italian medical schools highly, confesses to a grudging admiration for things Teutonic, and thinks that medicine has made advances in Italy and Spain. On the question of hobbies he speaks emphatically. "Every man ought to have a hobby. I am sorry for the man who has not got one. My own? Well, my hobby is everything that smacks of the sea; as a native of Liverpool my tastes are essentially marine. I was assistant surgeon in the Navy, and I have always retained a liking for things marine. My youngest son is a naval cadet. I have been to India, and six times to the United States, to Canada and Cali-

* An account of this interesting operation will be found in Symes' "Observations in Clinical Surgery," p. 170. The patient, who had been stabbed in the buttock seven years before the operation, recovered perfectly.

fornia, and up in the North Polar ice, and all over Europe and North Africa. Mountaineering? No, I cannot say I am a mountaineer, though, to be sure, I've been up the Peak of Teneriffe and some Swiss mountains."

### Early Days at Bart's.

With this interlude we hark back to purely professional things and the hospital world. Sir Dyce left Edinburgh and came to London, with a letter of introduction to Paget, of St. Bartholomew's. Here he became a student once more, and clerk to Sir George Burrows, working at his post-graduate studies—for he already possessed the M.R.C.S. diploma. "Then, when my term as resident in the college had expired, I returned to Edinburgh and took my degree, becoming a resident physician in the clinical wards of the Royal Infirmary for a year, went into the Navy, as my grandfather did, and served as assistant surgeon. When Kirkes' death created a vacancy at St. Bartholomew's I was advised to come back. I did so, and was made medical tutor. When we reorganised the nursing staff I was appointed lecturer in medicine to the nurses, an appointment which I held for nine years. Willett did the surgical nursing teaching. I had out-patient work for 15 years—five hours a day twice a week—also the skin department for four years. I was attached to the Royal General Dispensary, close to the Hospital, where I saw many interesting cases, worked hard with Sir William Church, and gained much good experience. It was a very valuable appointment, and I made full use of the opportunities it gave for medical study, necropsies, and management in the homes of the poor. I had a great deal of tutoring work to do as well."

From the date of his appointment as full physician to St. Bartholomew's, Sir Dyce Duckworth's history is well known to the profession, and his public services, not only in the capacity of examiner to the Admiralty, medical referee to the Treasury, and physician to insurance offices, but also as member of the General Medical Council and of certain great charitable funds, are too well known to readers of The Hospital to need detailment here.

### London and the Provinces.

Casting his mind back to those early years for purposes of comparison with the present, Sir Dyce finds much progress to record. "Things have improved marvellously in the provinces in recent years," he remarks. "There is good teaching—most excellent teaching, in fact—at the larger hospitals, and good discipline. But that, I think, was the case then. It is true, at the smaller hospitals work was done irregularly, of course with splendid exceptions. One found it out afterwards when one came to work with nurses, for instance, who had been trained in small infirmaries, apart from the discipline and regularity of a medical school, who were undisciplined and not to be depended upon. At St. Bartholomew's we always had a high ideal. Thus it was accounted bad form for the physician to be irregular or unpunctual in his duties; there was rigid discipline, and everything, private practice included, had as a rule to give way to hospital duty. It was sometimes difficult to adhere to this ideal, especially when one had to forego consultations to give hospital lectures; but we all felt that it was imperatively necessary if discipline was to be maintained. At present the discipline and teaching in the provinces are excellent, and I think the establishment of teaching universities has had a great deal to do in bringing this about. Also, you must bear in mind that there is far more public spirit—and, shall I say, sentiment—in provincial cities than in London. There is no sentiment here. London is a wilderness: no one loves London, and I go further even, and repeat what has been said of it, that 'London is the grave of public spirit.' A Liverpool man loves his town: he glories, exults in it. London, on the contrary, is kept up largely by men who come into it from other centres. The result of this is deplorable, and I am not exaggerating when I say that there is no sentiment here. That is one of the causes of the loss of endowments and the want of support of our medical schools. You are well aware that the provincial schools are attracting men. Why? Simply because they secure good teachers and appreciate them when they get them. They give good stipends and pensions. And we in London! We teachers have been paying largely for the education of the practitioner—we are doing so still. Just think for a moment what that means. At St. Bartholomew's the medical school is largely supported by the staff: at Guy's and other schools you know it is just the same. It is really grotesque when you come to consider it. Do you hear of lawyers doing that? Just fancy asking Government for a grant towards a metropolitan medical school, or appealing to the public! Then there is the University of London. Does anybody love it, or regard it tenderly as an *alma mater?* It may be—it ought to be—the greatest university in the world, but I doubt if I shall live to see it attain that proud position. Professor Gairdner used to say the present university simply prevents London getting a proper university, and he was quite right."

### The Way Out.

After this diatribe against the University of London, the natural query that suggests itself is: "How would you mend it?" But Sir Dyce is not merely a destructive critic: he has considered the remedy as well, and is equally emphatic about it.

"The material is there to work with. What is wanted is more public spirit, more healthy sentiment. It is only natural. The cost of living is greater here than in the provinces. Fewer students enter the profession, and yet it is overcrowded, and men are struggling hard in this keen competition for existence. The provinces have a pull over London because they have a better public spirit. Your provincial man of business is proud of his medical school and its teaching. He is willing to put his hand in his pocket and bear his share handsomely in its upkeep. That is not the case here. It is most disheartening appealing to a London man for practical support, simply because he does not know and does not care about the institutions, and takes little or no interest in scientific education in general, or medical schools in particular."

Dealing with medical students, Sir Dyce remarks that he has found the men educated at provincial schools very good. "They are keen men, and do well—some exceptionally well. Of course, we get the best of them, because the keen man usually finds his way to London for post-graduate work. But, speaking generally, I may say I have found provincial men usually better grounded in preliminary work—yes, sometimes better than a London University man. I am a great believer in extending study, and I should like to see provincial men come to London hospitals and London men go to provincial hospitals to get acquainted with the various methods. In general—and mind, I am speaking in general terms only, I have no particular case in mind—the purely London student is rather apt to be narrow and prejudiced. He knows only his school and hospital, and naturally regards it as the best. The Scottish student is a very hard worker. I think the climate has a great deal to do with that. The whole *genus* medical student is improving very much, and my own impression is that he deserves much more sympathy from the public than he gets. He is the best of all students—quite the best. That is exactly contrary to the common view? No doubt, but I speak deliberately. I have had to do with medical students all my life, and I speak about what I know when I say that, taking them as a class, you can find no more gentlemanly, clean-minded set of young men in any rank of life."

### The Curriculum.

There are few matters upon which the opinions of the heads of the profession are more instructive or more valuable than the question of the medical curriculum. On this point Sir Dyce has very decided opinions, and he has no hesitation in expressing them.

"You must look at it from the point of view of the average practitioner," he says. "For him it is a question of money and time. He does not want his son to be a 'specialist.' and I think we all agree that the education of the hospital teachers must of necessity be different from that of the student who desires, when he is qualified, at once to enter general practice. The latter should not be too elaborately taught, if I may so express it. To-day, I am sure, we waste too much time over abstract matters. We teach many of our students many scientific details which are of little value to them afterwards. Fifty years ago the teaching was much better in these preliminary sciences, because it was more practical. The application of chemistry, botany, materia medica, etc., was taught—and, bear in mind, it was then taught by men in practice—more than the scientifically interesting details of these studies. There is a notion that if the student is taught one or two subjects thoroughly he will be able to know the others. Just look at the modern teaching of physiology for the average student, can anything be more unpractical? Men are taught theories, and are expected to master them in minute detail: they are shown instruments, and expected to be familiar with the working of many of them which they will never possess or perhaps see again.

### Apathy in Practical Subjects.

Then contrast this energetic physiology teaching with the apathy shown in the teaching of other subjects of greater practical importance. There's materia medica—why, the average student is little familiar with his drugs, and he can seldom write a decent prescription! I made a point of getting my clerks to write out their prescriptions, and when I went round again there were the remedies above the patient's bed. Then I used to tell them to taste their mixtures—and very nasty they were sometimes, too. 'That is your fault,' I said. 'It's your prescription. Let me tell you that if that patient was a lady in Belgravia, and you had ordered her that mixture—well, she wouldn't have come back to you. You have come here to learn the work of your future life, and I intend that you shall learn it. You have not come here to learn how to be a physiologist or a chemist, but to become a general practitioner.' And I made them do everything. They had to learn how to bleed, how to make and apply poultices, make arrowroot, make beds, and, in fact, all the details of practical bedside work which are so much neglected nowadays. I feel very strongly on this point—the imperfect way in which therapeutics and materia medica are often now taught. The average young practitioner is not seldom now the laughing-stock of the dispensing chemist: his prescriptions, when they are not for tabloids, are often ridiculous, and the chemist knows it."

### Therapeutics and the Modern Student.

After this frank expression of opinion, he proceeds to give in detail the counts of the indictment against the modern teaching of therapeutics, and every general practitioner will in the main be in complete agreement with him. "The student of to-day is taught a dislike of opium; when he is qualified and in practice he is frightened of this drug and hardly ever uses it—until experience proves to him its great value. He thinks he ought never to use opium in abdominal cases because the surgeons tell him not to use it. The fact is he does not know how to use the drug. When he employs it he abuses it: he injects morphia subcutaneously. The new practitioner has come to disregard almost entirely the internal administration of opium. It is very much the same with other drugs. The good old tinctures and infusions, which after all are pre-eminent, are being displaced by tabloids and tablets. Calomel, I am glad to see, is coming back into professional favour. I wish one could say the same of bleeding and alcohol. Let me tell you this, that I think there are few drugs equal to good port wine. It is of infinite value when you know how and when to employ it, but that is just what the average student does not know. He is taught sometimes to look upon alcohol as "the devil in solution." Since wine has been so generally given up we have had more strange views in medicine than ever before. The level-headed man employs a little wine, and appreciates it; he is able to see its good, and rightly to estimate its value. The 'cranks,' who talk all sorts of nonsense about food and medicine certainly seem to lose the *mens medica*. All this is very bad for the profession, and very much to be regretted. Anyone who dares to put in a good word for alcohol nowadays is likely to be accused of ignorance and improper practice. This question of drugs is an important question, and one of the saddest things to me is to find that so little attention is paid to it. It is true enough the average patient is well-treated, but what I mean is that the niceties of prescriptions and prescribing are ignored—hardly even known. Even the consultants are often in fault in this respect.

"There have been great strides made in diagnosis, but I think the old rule holds to-day, as it did in the 'sixties, that more mistakes are made in medical diagnosis through want of care than through want of knowledge. A man cannot properly see three or four cases—bad, complicated cases—in a very short time. He cannot give them all the attention they want, and the odds are that he makes some mistakes. To hurry over one's case is hopeless. A consultant in a hurry is an abomination, and does not deserve his fee."

### Hospitals and the State.

Then the talk turns on hospitals and hospital, and it is refreshing to find that Sir Dyce is as outspoken on this question as on the subject of the medical teaching of the day. "I hope the day will never come," he exclaims, walking up and down his consulting room, "when the general hospitals are under State control. I am a staunch upholder of the voluntary system. Let the public support good hospitals, not little private institutions. The King's and the Hospital Sunday and Saturday Funds are doing excellent work, but I think people should look after the hospitals in their particular neighbourhoods better than they do at present. Then there seems to me to be a real need for pay hospitals—I mean small, if you like *special*, hospitals for people who can afford to pay a small amount towards their medical treatment. I cannot say I am in favour of pay wards in general hospitals, for I think it is a bad plan to mix the voluntary and pay systems. I see no harm in having a special paying ward—as already exists at some hospitals—but it should be entirely separate and have little connection with the general hospital."

### The Future.

Looking ahead, for one who can look backward upon so many years of progressive achievement, is as instructive as the relation of past experience, but Sir Dyce refuses to be drawn into the ranks of the prophets, though he is not unwilling to give a hint or two. "The tendency," he remarks, in conclusion, "is towards an excess of specialism"—probably he has seen Mr. Corner's strenuous attempt to elevate andrology to the rank of a definite specialty! "The all-round man is getting rare, and there is a great want of level-headed men. There is too much refinement, and men are apt to forget that a good general grounding is imperative, and that everyone must have a sound general knowledge before he starts specialising. A good so-called "abdominal" man must be a good general surgeon to be a safe man. I think gynæcologists should be primarily surgeons: they belong more to the surgical side than to our College. With regard to treatment, I think we are open to improvement in our means of assuaging pain. Of course, in rheumatic fever the salicylates have done wonders, but I think it is not so generally known as it ought to be that by this very alleviation of pain in rheumatic cases we are running some risks. The practitioner has a great responsibility in a case of early rheumatic endocarditis, for instance. Under salicylates the pain is quickly relieved and the patient gets better; the friends cannot understand why you want to keep the patient long in bed. The practitioner sometimes gives in to them and lets the patient get up. That is a great mistake, and I am always insisting upon it being a mistake. You must keep your patient in bed until you are absolutely certain that he can get up with safety, else you run a risk of letting him remain for life with a cardiac lesion. We have much to learn still in many subjects—arthritis, for instance, and nervous and psychical disorders. Then there is the vast field of cancer research and malaria. I am not absolutely certain that we have got the whole truth about malaria in the mosquito theory: we want further research in this subject.

### Serum Treatment.

Everyone is keen on serum treatment, which is regrettable in some ways, for the public has got quite an exaggerated idea about the matter. Only the other day I saw an old lady with a large uterine tumour, diabetes, and double pneumonia—as bad a case as you can come across—and the husband wanted to know if it was not advisable to try serum treatment for her! We ought to be cautious with these sera; they are not always harmless, and their use demands much discrimination. We have to bear in mind that modern research as to blood conditions, etc., and expert opinions upon clinical pathology are, as they must be, very costly requirements for many patients. If not conducted by experts they are of small value and apt to be quite misleading. They are often essential for accurate diagnosis, but it may be that they are sometimes not quite so necessary as they are often thought to be, and it appears that our younger men are rather apt to resort too speedily to these aids without bringing their own clinical acumen and wits to bear at once and persistently on all the features of the cases before them.

"It comes to this: who, in these days, is alone conceived to be sufficient for all the clinical requirements demanded of him? It would seem that a modern ordinary patient would require to be handed over to a company of experts before he could be efficiently dealt with. We must try, and try hard, in this twentieth century to be level-headed men, and to see all matters medical in due proportion, and seek to have the mind of some of the great clinical masters who have preceded us in our calling. They were not unwise men."

